# Unraveling the Mechanisms of Diabetic Wounds: Insights into Pathogenesis and Advanced Treatment Strategies

**DOI:** 10.2174/0115733998368222250509185511

**Published:** 2025-06-10

**Authors:** Nur Shaid Mondal, Aminul Islam, Manish Kumar Gautam, Sandip Mondal, Muskaan Ahmed, Sungjemrenla Jamir, Larity Daniewkor Khongwir, Bidhan Mahajon

**Affiliations:** 1 Faculty of Pharmaceutical Science, Assam down town University, Sankar Madhab Path, Gandhi Nagar, Panikhaiti, Guwahati, Assam, 781026, India;; 2 Department of Pharmaceutical Chemistry, Rahman Institute of Pharmaceutical Sciences and Research, Tepesia, Kamarkuchi, Assam, 782402, India;; 3 Amity Institute of Pharmacy, Amity University, Kolkata, West Bengal, 700135, India;; 4 Pratiksha Institute of Pharmaceutical Sciences, Barchapari, Guwahati, Assam, 781026, India;; 5 Institute of Pharmacy, Assam Don Bosco University, Tapesia Gardens, Sonapur, Assam, 782402, India;; 6 Central Council for Research in Ayurvedic Sciences, Ministry of AYUSH, Government of India, New Delhi, 110058, India

**Keywords:** Diabetic wound healing, matrix metalloproteinases (MMPs), chronic hyperglycemia, regenerative therapies, angiogenesis, gene therapy, stem cell

## Abstract

Diabetic wounds are a class of chronic wounds that exhibit significant healing abnormalities due to dysregulated cytokines, growth factors, and unique cellular expressions, currently affecting an estimated 9.1-26.1 million people per year globally. Matrix metalloproteinases (MMPs), angiogenic factors, and inflammatory mediators remain the key determinants for managing diabetic wounds. Vascular endothelial growth factor (VEGF) is one of the most prominent types of growth factors induced during angiogenesis in general and cell proliferation pathways. Chronic hyperglycemia, neuropathy, and inflammation associated with diabetes disorders affect cellular responses, blood circulation, and immunological systems impair normal wound healing. This reduced effectiveness of current management strategies is reflected in the high number of delayed wounds among diabetic patients due to escalated oxidative stress and impaired signaling pathways, which prevent healing, calling for new therapies. MMPs are essential for tissue remodeling, but excess levels of MMPs predispose tissues to matrix degradation and interruption in cell signaling leading thereby prolonging inflammation seen in diabetic wounds. Efficient wound healing requires a balanced relationship regarding matrix metalloproteinases and tissue inhibitors of metalloproteinases (TIMPs). New regenerative solutions, such as stem cells, platelet-rich plasma (PRP), gene therapies, and MMP inhibitors that can re-establish angiogenesis; decrease inflammation; and stimulate growth factor signaling, suggest promising strategies for improved diabetic wound healing. Hyperbaric oxygen therapy allows tissue regeneration and reduces the area of ulceration, bringing other benefits. In the future, therapeutics should focus on multifunctional and responsive strategies that include anti-inflammatory agents, cytokine modulators, and stem cell treatments that exhibit superior efficacy in comparison to conventional therapies when assessed clinically. Novel advanced combination strategies represent a realistic route to targeted therapies that meet clinical needs and have the potential capability for utilizing mechanistic insights, both creative in their implementation of recently developed techniques as well as applied on a broader scale through the evidence-based management across diabetic wounds offering better outcomes and quality of life amongst increasing diabetic commonalities.

## 1 INTRODUCTION

In terms of surface area, the largest organ of the human body is the skin. It is very important for protecting the inside tissues from extreme temperatures, ultraviolet radiation, microbial infections, and mechanical harm. Because of this, it is extremely prone to wounds, which can have a serious effect on specific people [1]. A wound is caused by the breakdown or destruction of the natural structure and functioning of bodily tissue. It originates from pathological processes that begin internally or externally in the affected organ or tissue [2]. Wound healing is a fundamental physiological function that involves the various cell strains and their products [3]. As a normal biological process in the human body, the healing of wounds is accomplished through four major phases. These four key phases of wound healing are the hemostasis phase, the inflammatory phase, the proliferative phase, and the remodeling phase. For a wound to properly and successfully heal within the allotted time frame, all four highly regulated phases are necessary [4].

Hemostasis is the initial phase of wound healing characterized by cessation of bleeding following the vascular injury. It lasts for two to three hours, during which time the aggregated platelets release growth factors and cytokines and create a fibrin plug [5]. It consists of three other sub-stages, *i.e.*, vasoconstriction, primary hemostasis, and secondary hemostasis. Vasoconstriction of the artery walls occurs instantly to stop bleeding when the skin is injured. The vasoconstriction is followed by primary hemostasis. During this stage, the platelets aggregate and form a temporary fibrinogen clot to avoid excess bleeding. After the primary hemostasis, the secondary hemostasis process activates, transforming soluble fibrinogen into insoluble fibrin mesh strands. Both the platelet plugs and fibrin mesh aggregate to produce a thrombus that inhibits bleeding [1].

Inflammation is the second phase of wound healing, characterized by an increase in the permeability of capillaries and migration of cells into the site of the wound [6]. It is also marked by chemotaxis, hemostasis, and enhanced vascular permeability, which helps to seal the wound, eliminate bacteria and cellular debris, promote cellular migration, and prevent further harm. The inflammatory phase typically lasts for a few days [7].

The inflammatory phase is followed by the proliferative phase. The proliferative phase is a multifaceted process that involves granulation tissue formation, angiogenesis, collagen deposition, epithelialization, and wound retraction [8]. After wounding the skin or tissue, the last phase, *i.e.* Remodeling phase, begins and might last for up to a year [9]. During this wound healing phase, fibrin clots, which are formed during the inflammatory phase, are replaced by type III collagen-rich granulation tissue and blood vessels during the early inflammatory phase. This is then followed by the replacement of a collagenous scar made of type I collagen with less developed blood vessels, resulting in a more efficient healing process [10].

Also, there are significant health risks and problems with the quality of life for those whose wounds do not heal within a normal period, and because of chronic health issues and aging populations, the amount and frequency of nonhealing wounds are rising [11]. This may include vascular insufficiency, radiation therapy, chronic diseases, immunosuppression, age, body type, and nutritional state [12]. However, diabetes mellitus (DM), a metabolic condition, is one of the major causes of delayed and impaired healing of wounds. DM is a metabolic disorder that has been characterized by hyperglycemic conditions. Both acute and chronic wound healing are impacted by impaired healing in diabetic conditions [13].

Chronic hyperglycemia can lead to long-term harm, malfunction, and failure of various organs such as the heart, kidneys, eyes, blood vessels, and nerves [14]. One significant diabetic consequence linked to higher rates of morbidity and death is impaired wound healing. Amputation is the most common outcome for nonhealing wounds, which raises the direct expenses of management, recovery and reduces productivity [15]. The high rates of incidence, disability, and death have made diabetes a global health concern. It is the eighth most common cause of death and disability worldwide. The yearly cost of treating diabetes in adults aged 20 to 79 was anticipated to be US$ 966 billion in 2021 and is expected to rise to over $1054 billion by 2045 [16]. The age-standardized diabetes prevalence is 6.1% worldwide, up 90.5% from 3.2% in 1990, and is expected to rise to 9.8%, affecting 13.1 billion people. According to International Diabetes Federation reports, globally, 537 million individuals had diabetes in 2021, and a diabetic patient had a 25% lifetime risk of developing foot ulcers [17, 18]. Diabetic foot ulcers (DFU) are open wounds experienced by 15-25% of diabetic patients, with a global incidence rate of 9.1-26.1 million per year [19]. In recent years, the number of people with diabetes has drastically increased in nations like India. Globally, India is known to have the largest population suffering from DM, and it is also estimated that by 2025 the numbers are expected to increase much more [20]. It is estimated that fifteen percent of persons with diabetes have diabetic foot ulcers (DFU), out of which 14-24% go on to have a lower extremity amputated. Additionally, it is shown that the amputation-related death rate in diabetes patients increases by 50-59% five years following the procedure [21].

## 2 METHODOLOGY

### 2.1 Search Strategy

We conducted a comprehensive search using the terms “diabetic wound,” “hyperglycemia,” “gene therapy,” “stem cell,” and “healing mechanism” across the following language-neutral electronic databases: Medline, EMBASE, AARP Ageline, AMED (Allied and Complementary Medicine), The Cochrane Database of Systematic Reviews, DARE (Database of Abstracts and Reviews), and Google Scholar.

### 2.2 Inclusion Criteria

This review encompasses studies on the healing mechanisms of diabetic wounds and diabetic foot ulcers, as well as those concentrating on hyperglycemia. Research on the potential therapeutic applications of regenerative medicine, stem cell therapy, and gene therapy for diabetic wound healing was also considered. Only articles from peer-reviewed publications, clinical trials, and systematic reviews were evaluated. Research on the treatment of diabetic wounds may utilise *in vitro*, *in vivo*, or clinical models; however, it must be published in English.

### 2.3 Exclusion Criteria

Studies were excluded if they exclusively addressed wounds that were not diabetic or if they had no bearing on wound healing or diabetes. Studies with insufficient data and duplicate publications were not included. Articles written in languages other than English, as well as studies with no clear methodology or results relevant to gene or stem cell therapy, were also not taken into account.

## 3 MECHANISTIC INSIGHT

The pathophysiology behind the impaired wound healing in diabetic patients remains unclear [22]. Numerous extracellular elements, cells, growth factors, and cytokines are all crucial for all four phases of wound healing, but in chronic disorders, especially DM, impair wound healing by interfering with these normal biological systems. The most common causes may include hypoxia, autonomic and sensory neuropathy, hyperglycemia, long-term inflammation, abnormal micro- and macrocirculatory function, and compromised neuropeptide signaling [23]. When an injury of tissue occurs, an inflammatory process takes place because neutrophils, macrophages, and mast cells produce several kinds of growth factors, such as epidermal growth factor (EGF), platelet-derived growth factor (PDGF), fibroblast growth factor (FGF), vascular endothelial growth factor (VEGF), transforming growth factor (TGF) and insulin-like growth factor 1 (IGF-1), as well as inflammatory cytokines, including tumor necrosis factor-alpha (TNF-α), interleukin 1 (IL-1), interleukin 6 (IL-6), and interferon-gamma (IFN-γ). These growth factors are vital for the wound-healing process [24]. Individuals with DM have an imbalance of these cytokines, which alters the wound healing process [25]. Additionally, it has been documented that neutrophils exhibit a changed pattern of cytokine release and a reduction in functioning, leading to increased risk of potential wound infection [5].

Research also suggests that by inducing the synthesis of matrix metalloproteinases (MMPs), neutrophils play a vital role in the development of a degradative product that leads to an unbalance between matrix production and breakdown [26]. Extended neutrophil infiltration is also linked with increased elastase, reactive oxygen species (ROS), and reactive nitrogen species (RNS) in the wound area, these entities possess notable cytotoxic and pro-degradative capabilities [27]. The cytotoxic cascade of diabetic wounds, especially diabetic foot ulcers, is exacerbated by a pro-oxidant environment that complements the pro-inflammatory aspect [28].

Following an injury, the wound becomes hypoxic due to damage to blood vessels [29]. Hypoxia decreases oxygen levels, leading to hemoglobin glycation, causing insufficient oxygen and nutrition delivery to the wounded site of the tissue, which further slows down the healing process. The cell's stress response is triggered by hypoxia/glucose shortage, distorted proteins, and the unfolded protein response (UPR) within the endoplasmic reticulum [30]. UPR is associated with the synthesis of pro-inflammatory cytokines and is triggered soon after tissue damage. Diabetes-related wounds exhibit prolonged induction of UPR and increased production of pro-inflammatory chemokines, compared to normal wounds, indicating a potential link between tissue or skin damage [31].

The release of VEGF by fibroblasts, macrophages, and epithelial cells (EPC) during wound-induced hypoxia triggers the phosphorylation and activation of eNOS in the bone marrow. It raises NO levels, which in turn causes the mobilization of bone marrow EPCs into the circulation. These EPCs are guided to the injury site by the chemokine SDF-1a, where they take part in neovasculogenesis [32].

Hyperglycemia causes atherosclerosis and impaired functioning of various skin cells has a direct impact on wound healing and the development of DFUs [33]. Hyperglycemia elevates atherosclerosis, which limits the passage of nutrients to the damaged tissues and slows the healing process [34]. Additionally, patients with DFUs exhibit abnormal expression of keratinocyte proteins important for re-epithelialization. These include cytoskeletal keratin proteins (K2, K6, and K10), crucial for the keratinocyte differentiation process, and laminin-5 α3 precursor protein, which regulates epithelial cells' binding to the basement membrane [35].

Endothelial cell abnormalities are also a major factor causing impaired wound healing in diabetic individuals. Compared to persons without diabetes, patients with diabetes have higher rates of macrovascular disease, which is more distal in its distribution from the superficial femoral artery to the pedal arch and involves the metatarsal artery [36]. Autonomic and sensory neuropathy also contributes to impaired healing of wounds [23]. Neuropeptides like substance P, nerve growth factor, and calcitonin-related peptides are crucial for wound healing by increasing cell chemotaxis, promoting growth factor production, and driving cell proliferation. The occurrence of DFUs in diabetics has been connected to a reduction in these neuropeptides. Sensory nerves also play a crucial role in immune defense processes, and denervated skin exhibits a decrease in leukocyte infiltration [4]. Unlike diabetic nephropathy, diabetes causes a reduction in angiogenesis in wound healing. Diabetic wounds have reduced vascularity and capillary density due to inadequate angiogenesis (Fig. **1**) [21].

## 4 ROLES OF FREE RADICALS AND OXIDATIVE STRESS

Oxygen is crucial for life and biological systems, but its reduction in cells produces free radicals, which can cause damage to tissue through oxidative stress. The generation of free radicals occurs in various cellular locations inside the cytosol, plasma membrane, endoplasmic reticulum, mitochondria, and lysosomes. In these sites, free radicals are generated from various endogenous and exogenous sources [37]. The reactive oxygen species (ROS) and nitrogen species (RNS), which are formed from cellular metabolic processes mainly contribute to the generation of free radicals. Besides reactive species, there were various non-reactive species are there which were also responsible for free radical formation [38]. ROS, including hydroxyl radical, superoxide anion, and hydrogen peroxide, are crucial for generating free radicals, and excessive production can result in oxidative stress [39, 40]. Oxidative stress damages biological structures like proteins, lipids, and nucleic acids, potentially leading to chronic diseases like diabetes, atherosclerosis, metabolic diseases, cancer, and heart disease [41].

High levels of oxidative stress are a significant contributor to diabetes-related issues and delayed healing. Increased production of ROS within cells due to high glucose levels triggers intracellular metabolic pathways, including neuropathy, the protein kinase C (PKC), hexosamine, polyol pathways, and advanced glycation end products (AGEs) formation, which in turn suppress antioxidant enzymes [42]. Furthermore, studies also revealed that Nrf_2_, a transcription factor that is crucial for wound healing through antioxidant gene regulation and angiogenesis, is also impaired in diabetes [42, 43].

The stress-triggered gene ATF-3 causes B-cell malfunction and diabetes implications. It is activated due to an unusual pro-inflammatory response that disrupts redox equilibrium and hampers healing pathways [44, 45]. In diabetic patients, excess glucose reacts non enzymatically with an amino acid's NH_2_ group, altering protein function and causing the production of AGEs [39]. Recent studies revealed that diabetes also leads to elevated levels of serum AGE and its receptors, which bind to endothelial cell surface receptors, causing overproduction of ROS, which leads to impaired wound contraction in diabetic conditions [46, 47]. AGE appears to negatively impact the proliferation of extracellular matrix (ECM) and prolongs the inflammatory responses [42].

The polyol pathway, which turns glucose into sorbitol and fructose, is a key mechanism in diabetes. Elevated glucose levels increase the affinity of aldose reductase (AR) and sorbitol dehydrogenase for glucose, leading to increased sorbitol production. This accumulation reduces Na^+^K^+^-ATPase activity, reducing nerve cell physiological reserve and conduction velocity [48]. The polyol pathway induced by hyperglycemia increases oxidative stress due to depletion of NADPH and excess fructose, accelerating glycation and exacerbating intracellular oxidative stress. Diabetic mice with high aldose reductase expression show significant sorbitol accumulation in the sciatic nerve, causing oxidative stress and DNA damage [49]. AR inhibitors are utilized to inhibit oxidative stress *via* the polyol pathway [50].

Hyperglycemia activates the hexosamine pathway *via* fructose-6-phosphate, causing impaired nerve function and increasing the production of cytokines like TGF-α and TGF-β [51-53]. This pathway leads to increased Sp1 transcription factor, PAI-1, and TGF-β1 overexpression in endothelial cells, and increased thrombospondin 2, causing delayed wound healing [54].

PKC is a protein kinase that affects various cellular signaling pathways, and its isoforms, including PKCα, βI, βII, and δ, are very significant for peripheral nerve conduction [55]. Hyperglycemia leads to the overproduction of oxygen free radicals, which inhibits glyceraldehyde phosphate dehydrogenase activity, accelerates DHAP conversion to DAG, and activates the PKC pathway [56]. This will lead to endothelial dysfunction by reducing NOS and impairing healing through increased levels of endothelin-1 and ROS [57]. Studies indicate that nonselective PKC isoforms or selective PKC-δ inhibitors can enhance nerve function in diabetic rats, potentially offering a new method for treating diabetic wounds [58]. Diabetic peripheral neuropathy, a condition involving sensory, motor, and autonomic dysfunction, can cause delayed wound healing. Neuropathic skin has reduced neuronal density and slower healing rates. Sensory neuropathy can lead to diabetic wound growth. Hyperglycemia and oxidative stress lead to abnormal glycation of nerve cell proteins, resulting in nerve dysfunction and ischemia [59, 60] (Fig. **2**).

## 5 IMMUNE SYSTEM

The immune system plays a significant role in the wound healing process. An imbalance of immune cells can stall the wound in the inflammatory phase, which hampers further wound repair processes [61]. Numerous inflammatory cells, such as mast cells, T cells, B cells, neutrophils, and monocytes, are crucial for developing immunity [22].

Macrophages from the monocyte play a crucial role in wound repair [62]. Macrophages are phenotype-based and divided into activated macrophages (M1) and 'M2' subgroups. 'M1' macrophages play a role in pathogen elimination, pro-inflammatory cytokine release, and wound repair processes. Conversely, 'M2' subgroup macrophages are involved in the remodeling of extracellular matrix, angiogenesis, wound healing, and anti-inflammatory cytokine generation [63]. M1 macrophages are polarized by pro-inflammatory cytokines like tumor necrosis factor (TNF), lipopolysaccharide (LPS), and interferon, leading to generate interleukin (IL) 12, IL-23, NO, as well as ROS [64]. However, M2 macrophages are polarized by anti-inflammatory cytokines like IL-4, IL-10, and IL-13 polarize M2 macrophages [64]. A balanced M1 and M2 polarization is in keeping with normal wound healing [65]. M1 macrophages predominate over the first three days of normal wound healing, and then after they transform into M2 macrophages, which gradually take over [66]. Hyperglycemic condition is one of the major pathways that leads to an increase in pro-inflammatory cytokines, which in turn increases the polarization of M1 macrophages. Elevated glucose stimulates macrophages to secrete pro-inflammatory cytokines, such as IL-1, IL-6, and TNFα, promoting a vicious cycle that maintains M1 macrophage polarization and chronic inflammation [67]. Thus, in diabetic conditions, the transition of M1 to M2 is impaired, leading to delayed wound healing. This impaired polarization of M2 in wounds with diabetic conditions is linked to low collagen deposition and impaired angiogenesis [68]. This impaired pro- to pro-to-anti-inflammatory macrophage transition halts the wound in the inflammatory phase, resulting in delayed wound healing [9].

The molecular pathway of M1/M2 macrophage polarization is not well defined, but the main linked pathways are interferon regulator (IRF), non-receptor tyrosine-protein kinase/signal transducer and activator of transcription (JAK/STAT), Notch, and phosphatidylinositol-3-kinase (PI3K/AKT). Long noncoding RNAs (lncRNAs) and microRNAs (miRNAs) also play a crucial role in macrophage polarization [67]. Additionally, M1 macrophage polarization can result from obesity alone by increasing plasma-free fatty acids, which causes the nuclear factor kappa-B (NF-κB) pathway to express more inflammation [69]. Autophagy regulates macrophage polarization, with rapamycin, an inducer of autophagy, activating the mammalian target of rapamycin (mTOR) pathway, thereby stimulating the M1 phenotype of macrophages. Furthermore, studies have also reported that through autophagy activation, AGEs promote the polarization of macrophages toward the M1 phenotype, leading to persistent wounds [70]. High hyperglycemia, local ischemia, and hypoxia in diabetic wounds enhance the number and proportion of M1-type macrophages, which leads to increased content of inflammatory factors. Thus, all the pathways hamper and hinder the transformation of M1 macrophages to M2 macrophages, resulting in excessive wound inflammation and delayed wound healing [67, 71]. In broad terms, the polarization of macrophages toward an anti-inflammatory type significantly improves delayed wound healing. Therefore, various approaches for modifying the macrophage phenotype can be used as therapies to treat chronic wounds.

Neutrophils, a crucial component of bone marrow stem cell-derived leucocytes, act as the initial defense against pathogens and are vital in the early inflammatory phase of wound repair. They phagocytose and degranulate cell debris at the wound site, ensuring efficient wound repair [72]. Neutrophils form a net-like construction called neutrophil extracellular traps (NET) when pathogens stimulate them. NETs are decondensed chromatin released from the nucleus of neutrophils coated with cytosolic or granular proteins, proteases, and histone, creating a net-like structure to kill microbes. This process of formation of NETs from neutrophils is called NETosis [73]. Neutrophil elastase (NE) is an enzyme found in the cytoplasmic granules of neutrophils. It enters the nucleus when pathogens stimulate and modify the histone proteins, and ultimately leads to chromatin decondensation [74, 75]. Elevated levels of NE are seen in the wounded area, which leads to the development of NETs, causing breakdown of the wound matrix and ultimately slowing down wound healing. Elevated NETs are responsible for delayed wound healing [73]. NET is also generated by a calcium-dependent enzyme, peptidyl arginine deiminase 4 (PAD4) [76]. For NET formation, calcium efflux is very crucial, as it produces ROS and PAD4-mediated chromatin decondensation [77]. The patient with diabetes has been found to have elevated levels of PAD4 expression and NE in their examined neutrophils. NETs are impaired by DNase, which could be a potent pathway to manage diabetic wounds [78]. Diabetes triggers neutrophils to overexpress the NETosis, PAD4, and NE, leading to elevated NETs and causing delayed wound healing [79]. Research indicates that receptors for the advanced glycation end products (RAGE)-AGEs pathway can hinder efferocytosis in diabetic wounds, inhibit AGE-RAGE activation, and increase neutrophil clearance in diabetic mice. It may be a potent target for managing diabetic wounds [65]. In diabetic patients, inhibition of PAD4 and DNase1 during the NETs' intervening process may be a crucial pathway for wound healing [79].

Dendritic cells (DCs) are diverse phagocytosis cells found in all tissues, expressing various pathogen identification receptors and regulating innate and adaptive immune responses to maintain tissue stability. Because of their high sensitivity, dermal DCs can identify infections or dead cells and send signals to effector T cells for monitoring [65]. T- lymphocytes are essential for the complete remodeling of granulation tissue that fibroblasts have laid down; without them, wound healing is slower and shows reduced integrity. By connecting innate and adaptive immunity, dermal DCs play a vital role in the immune system. Their main job is to transport antigens to CD8 and CD4 T cells [80]. DCs play a crucial role in clearing apoptotic cells during skin injury, with membrane proteins and transporters like SLC7A11 inhibiting abnormal cell proliferation. DCs have unique glycogen reserves in phagocytes, which can be inhibited by SLC7A11 due to its role in promoting efferocytosis. Inhibiting SLC7A11 function can accelerate wound healing in diabetes-prone mice [65].

Studies have shown that diabetic foot ulcer margins had lower amounts of CD3^+^ T-lymphocytes than non-diabetic people. Furthermore, diabetic wounds seem to have a low CD4^+^/CD8^+^ T-lymphocyte ratio. Another study also finds that diabetic individuals have more CD4^+^CD28 null T-lymphocytes, which release pro-inflammatory cytokines such as interferon-gamma and C-reactive protein [81]. These cytokines activate monocytes, contributing to the chronic inflammatory state, a finding not found in non-diabetic patients. T-Regulatory cells (Tregs), which control inflammation and avert needless immune reactions, are more prevalent in diabetic mice. Nevertheless, these Tregs have weakened immunoregulatory capabilities, which may cause diabetic patients' immune systems to malfunction. More research is required to ascertain how changed Tregs affect wound healing, particularly in diabetic wounds [65, 81]. Hyperglycemia affects γδ-T cells, a subset of T-lymphocytes that play a role in wound healing and surface barrier defense. A study on mice found that hyperglycemia decreases the proliferation and function of γδ-T cells, increasing vulnerability to infection, epithelial injury, and wound healing [81]. The chemokine receptor (CXCR3) ligand in the T cell subtype (Th1 cells) is highly expressed in chronic inflammation, and diabetic ulcer patients also show increased levels of inflammatory T cell subtypes, like Th1, Th17, and Th22 [80].

Elevated AGEs in hyperglycemia can also increase immunity by stimulating cytokine growth like IL-6 and TNF, preventing the formation of collagen, causing apoptosis, and hyperactive immunological response, leading to slowed wound recovery [82]. Mast cells, along with fibroblasts, macrophages, and endothelial cells, significantly boost wound repair by disturbing the balance of pro-angiogenic and anti-angiogenic molecules in damaged tissue [83]. Mast cell dysfunction in diabetes leads to slowdown in vascular regression in diabetic mice with a hereditary mutation [84]. This dysfunction affects angiogenesis during the proliferation, vascular regression, and remodeling phases, as they release potent angiogenic agents like FGF, VEGF, and TGF-β1 [85]. Tellechea *et al.* found that increased mast cell degranulation on the skin surface slowed healing in humans and mice. Blocking mast cell degranulation with quercetin, luteolin, and disodium cromoglycate may improve wound recovery in diabetics [83]. This suggests that mast cell degranulation inhibition can be a potential diabetes treatment. Diabetes leads to a decline in MIF gene expression, a key component of the innate immune system's proinflammatory responses. This deficiency in DWs leads to decreased endothelial progenitor cell generation and delayed wound recovery. Heat shock proteins (HSPs) play a crucial role in wound recovery by promoting cell proliferation, recruiting fibroblasts, reducing oxidative stress, and promoting protein homeostasis. The decrease in HSP levels in diabetes may contribute to delayed recovery (Fig. **3**) [86, 87].

## 6 GROWTH FACTORS

Wound healing is a complex process involving hemostasis, cell migration, maturation, inflammation, ECM deposition, angiogenesis, and remodeling. It promotes angiogenesis, proliferation, and chemotaxis *via* interacting with different cells, growth factors, and enzymes [88]. During normal injury, M1 macrophage transforms into M2 macrophages by recruiting cytokines and growth factors, causing endothelial, keratinocyte, and fibroblast cells to proliferate, differentiate, and migrate [89]. The generation of ECM and clotting is crucial for angiogenesis after injury. Growth factors like VEGF, PDGF, EGF, IGF-I, IGF-II, TGF-β, KGF, and FGF play crucial roles in wound healing stages, including stimulating granulation tissue, modulating inflammation, promoting angiogenesis, and remodeling ECM [22, 90, 91], thus dysregulation of them often results in impaired wound healing.

VEGF is a growth factor that stimulates vasculogenesis and angiogenesis and is released during wound healing by platelets, macrophages, and keratinocytes [92, 93]. It is also crucial for various functions such as endothelial cell proliferation and migration, epithelialization, vessel permeability, collagen deposition, ECM degradation, and vasodilation [94, 95]. VEGF enhances the density of capillaries in wounds with diabetes, promotes perfusion of blood, and promotes wound healing by restoring the flow of blood to wounded tissues. It regulates revascularization and granulation tissue formation, with activation of receptors affecting inflammation and angiogenesis [22]. VEGF expression is high in diabetic individuals as compared to healthy ones due to oxidative stress generation in hyperglycemic conditions [96, 97]. Studies showed that diabetic mice experience reduced expression of VEGF and its receptor VEGF receptor-2 in wounded keratinocytes and other skin tissues [98-100]. Hypoxia developed in normal wounds activates hypoxia-inducible factor-1 (HIF-1) to promote wound healing. Various factors are upregulated by HIF-1, but mainly, VEGF expression is upregulated. But hyperglycemia inhibits the HIF-1, thus downregulating the VEGF expression, resulting in aberrant normal cellular and ECM function and reducing angiogenesis in diabetes-related wounds [101-103].

EGF is primarily produced by platelets, macrophages, and monocytes, which bind with epidermal cells and fibroblasts to produce their effects [104]. EGFs also promote cellular migration, re-epithelialization, ECM remodeling, cellular proliferation, and angiogenesis [105, 106]. However, studies revealed that the levels of EGF are reduced in diabetes-related wounds, with most EGFRs being migrated to the cytoplasm instead of the membrane of the cell [32, 107]. Clinical trials have demonstrated that the application of topical EGF to diabetic wounds enhances epithelialization and accelerates healing [108, 109]. Clinical trials also show that recombinant human EGF (rh-EGF) can prompt healing in diabetic foot ulcer patients [110]. Heberprotp^®^, Regen-D^TM^150, and Easyef^®^ are commercially available drugs containing rh-EGF [106].

Platelets are the first responder that gets into the wounded area and releases various growth factors, along with PDGF, which proliferate and migrate inflammatory cells, including neutrophils, monocytes, smooth muscle, and fibroblast cells to the wound area [103, 111, 112]. It significantly promotes extracellular matrix production, stimulates fibroblast differentiation, and facilitates collagen contraction, glycosaminoglycans, proteoglycans, granulation tissue proteins, and angiogenesis during the proliferation stage of the healing process [22, 111]. PDGF can interact with PDGF-R receptors on fibroblasts and smooth muscles, thereby promoting neovascularization and re-epithelialization [103, 113]. Studies have proven that PDGF and its receptor expressions are significantly decreased in wounded diabetic db/db mice and glucocorticoid-treated mice [114, 115]. TGF-β can stimulate PDGF gene expression, but hyperglycemic conditions reduce TGF-β levels, potentially downregulating PDGF in diabetic wounds [116]. Several clinical studies also showed that PDGF can improve impaired wound recovery in diabetic individuals [117]. The PDGF-BB and PDGF-D phenotypes were potent in improving wounds in diabetic and ischemic patients [118, 119].

The FGF plays a crucial role in wound repair, primarily involved in re-epithelialization, stimulates keratinocyte proliferation, granulation tissue generation, ROS detoxification, and matrix formation [103]. Basic fibroblast growth factor (bFGF or FGF2), a phenotype of FGF, stimulates fibroblast growth, increases granulation tissue generation, endothelial cell proliferation, extracellular matrix metabolism, mesoderm cell growth, and promotes angiogenesis [120]. It works synergistically with VEGF to promote angiogenesis and is also seen as upregulated in DFUs recovery processes. It is also associated with increased ERK1/2 protein levels, suggesting its potential in wound repair *via* the ERK1/2 pathway [93]. bFGF topical application enhances wound strength, closure rate, and restores angiogenesis in diabetic mice, reversing impairment in healing wound strength in diabetic rats [104].

The TGF-α superfamily, produced by cell types such as platelets, macrophages, neutrophils, and fibroblasts, regulates tissue homeostasis in multicellular organisms [103, 121]. TGF-β1 stimulates inflammatory cells, including growth factors, neutrophils, macrophages, fibroblasts, and keratinocytes, accelerating vascularization, angiogenesis, and ECM generation [22]. Studies show reduced TGF-β concentration in wound fluid of diabetic rats and humans with DFU have reduced TGF-β1 levels [116]. TGF-α receptors on different cells are also reduced in chronic diabetic ulcers. MMP genes have a TGF-α1 dependent inhibitory element, causing decreased TGF-α levels in wounds with diabetes, thus increasing MPP expression, leading to increased growth factor degradation [122]. TGF-α1 also activates transcription factors like Smad-2, Smad-3, and Smad-4 for collagen production, but in diabetic wounds, collagen synthesis is impaired due to TGF-α1 deficiency [123].

IGF-1 and IGF-2 peptides form a complex IGF, facilitating wound repair through cell granulation, endothelial cell migration, and re-epithelialization by activating Protein Kinase B (PKB). It also stimulates ECM production and acts as a mitogenic agent for fibroblasts and keratinocytes. In diabetes patients, decreased IGF-1 expression may cause cell granulation imperfection [22, 111]. IGF-1 alone or with other growth factors, enhances wound healing by enhancing collagen synthesis, cell proliferation, and angiogenesis [106].

Diabetes leads to decreased levels of nerve growth factor (NGF), which is crucial for wound healing, promoting cell proliferation, migration, angiogenesis, fibroplasia, and peripheral nerve regeneration [103]. Keratinocyte growth factor (KGF) plays a vital role in re-epithelialization and collagen deposition, but its expression is reduced in diabetic mice, which may potentially cause delayed wound healing [104, 111]. The deficiency of these growth factor levels and expression in diabetic conditions results in delayed wound healing.

## 7 ROLE OF PERIPHERAL ARTERY DISEASE AND VITAMIN D

Peripheral Artery Disease (PAD) is a serious consequence of diabetes, marked by the gradual constriction of arteries due to atherosclerosis, resulting in reduced blood flow to different parts of the body, organs, and tissues [124]. It is more common in those with diabetes and is more frequently attributed to hypertension, hypercholesterolemia, tobacco use, obesity, and diminished renal function. If untreated, PAD may result in serious consequences, including myocardial infarction and cerebrovascular accident. Diabetes aggravates PAD by hindering arteriogenesis, the formation of collateral blood flow. This dysfunction is exacerbated by hyperglycemia, insulin resistance, and metabolic diseases [125]. The molecular processes by which diabetes affects peripheral artery disease (PAD) encompass compromised shear stress detection, diminished expression of endothelial nitric oxide synthase, and heightened oxidative stress. The wound healing process in diabetes patients is distinct, resulting in infection and osteomyelitis, which may require amputation. Hyperglycemia-induced atherosclerosis and peripheral artery disease are variables that impede wound healing in diabetic individuals [116]. Hyperglycemia expedites atherosclerosis *via* advanced glycosylation end-products (AGEs), heightened oxidative stress, and dyslipidemia, resulting in inflammation, oxidative stress, and endothelial dysfunction. Peripheral artery disease (PAD) hinders angiogenesis, the process of new blood vessel development, and diminishes the generation of growth factors vital for wound healing. Comprehending the relationship between hyperglycemia-induced atherosclerosis and peripheral artery disease is essential for enhancing wound healing results in diabetic individuals [125].

Recent studies have highlighted the potential benefits of vitamin D in diabetic wound healing, particularly in the treatment of DFU. Vitamin D has been found to regulate inflammation, modulate immune response and oxidative stress, promote angiogenesis, and improve insulin signaling pathways [124, 125]. Vitamin D plays a crucial role in regulating insulin and glucose metabolism. Vitamin D increases the release of insulin from pancreatic β-cells by boosting cytoplasmic calcium levels, and it also enhances glucose uptake in skeletal muscle by regulating calcium concentrations and promoting the translocation of glucose transporter 4 [124, 126].

Findings from a systematic review suggest that vitamin D plays a crucial role in anti-inflammatory activities. Lower levels of serum vitamin D (SVD) reported in DFU are considered due to significantly higher levels of IL-6 as well as other inflammatory markers, namely CRP, ESR, CCL2, IL1b, TNF-α, and IFN-γ. It also reduces inflammatory responses by suppressing the production of pro-inflammatory cytokines and activation of nuclear factor-kappa α(NF-κα). It also significantly reduces oxidative stress by scavenging ROS [124]. Animal studies also suggest that SVD maintains the stability of NF-κα by inhibiting its activation [127]. Another *ex vivo* study suggests that supplementation of vitamin D may lead to downregulating the expressions of IL-1, IL-6, IL-8, and TNF-α [128]. Another animal study suggests that vitamin D significantly improved vascular endothelial function in diabetic wounds by increasing eNOS protein expression and inhibiting Vcam-1 mRNA expression. It also increased CD31-positive area, VEGF, VEGFR2, PDGF, PDGFRβ, bFGF, and EGFR expression, which promotes angiogenesis in diabetic wounds [125]. All these findings suggest that Vitamin D could be a potential agent to reduce inflammation along with other associated factors like neuropathy, PAD, neovascularization, endothelial dysfunction, and angiogenesis, which are significant contributors to diabetic wounds, particularly DFU [124].

Several studies also support that vitamin D may stimulate innate immune responses. The immunomodulatory properties of vitamin D and its ability to prevent inflammatory T-cell activation are well recognized through several studies. During infection, macrophages stimulate the production of 1,25-dihydroxy vitamin D3 by inducing the expression of 25-hydroxyvitamin D 1α-hydroxylase. This, in turn, stimulates the expression of genes that encode antimicrobial peptides [129]. Vitamin D also enhances the antimicrobial peptide production activity of epidermal cells, keratinocytes, and other cells [130]. Moreover, studies also suggest that vitamin D may regulate immune dysregulation by downregulating IL-1b and IL-6 expressions [131].

Vitamin D receptor (VDR) expression suggests that vitamin D is more than just a calcium regulator; it can act as a transcription factor that affects the immune system, regulates the inflammatory response, and can improve insulin sensitivity and function [132]. In diabetic patients, VDR is susceptible to developing complications of foot ulceration, derived from dysregulated systemic inflammation and infection. VDR has several single-nucleotide polymorphisms (SNPs) that can affect receptor functions, including those related to diabetic complications [133]. SNPs in VDR are thought to impact the activity of VDR. ApaI, a silent polymorphism, stabilizes mRNA and modulates vitamin D expression. FokI, along with ApaI, has been linked to vitamin D activity, indicating that ApaI and FokI SNPs are functional polymorphisms in terms of DFU and VDR activity [134].

## 8 ROLE OF MMPs IN IMPAIRED WOUND HEALING

Matrix metalloproteinases (MMPs) are essential enzymes in the wound repair process, belonging to the metalloendopeptidase family [135, 136]. MMPs are categorized into various subgroups such as collagenases, stromelysins, gelatinases, membrane-type MMPs (MT-MMPs), and others according to their structure [137]. MMPs destroy various matrix proteins, including collagen, basement membrane collagen, and elastin. Gelatinases, specifically MMP-2 and MMP-9, are primarily responsible for breaking down collagen type 4 from the underlying matrix [138]. They have a role in several phases of wound repair, including migration of cells through ECM proteolysis, re-epithelialization through the breakdown of junctional proteins, leukocyte invasion through the creation of a chemotactic gradient, and inflammation through the processing of several cytokines either through activation or inhibition [139]. They significantly influence growth factors availability, matrix structure, and cell surface signaling systems, which have an impact on apoptosis, proliferation, and differentiation of cells [140]. Enzyme MMP-1 is primarily responsible for the re-epithelialization, MMP-2 is involved in angiogenesis and matrix modeling, and MMP-3 is essential for wound contraction and remodeling [22]. MMP-9's role in healing is unclear, but it may help in separating keratinocytes from the membrane for migration and breaking down the matrix when necrotic or injured tissue is removed [141]. The activities of MMPs are regulated by tissue inhibitors of metalloproteinases (TIMPs) [142]. Research findings indicate that diabetic wounds are associated with elevated levels of MMPs, with chronic wound fluid levels over 60 times greater than acute wounds, which promotes tissue degradation and impedes healing [136]. TIMPs–MMPs complexation promotes effective wound healing through a balanced combination of TIMPs and MMPs by limiting MMP activity and preventing interference with the active site [143]. All these variables interfere with essential elements like growth factors and other proteins necessary for the wound to heal normally (Fig. **4**).

By promoting increased activity of MMP, senescent cells greatly contribute to the development of impaired wound healing in diabetes. The high activity level of active MMPs degraded not only major components of the ECM but also growth factors and cell surface proteins, creating a tissue environment unsupportive of repair. This further prolongs chronicity in inability by senescent cells to downregulate the activity of MMP, therefore pointing to the need for therapies that target both MMP and senescence [144].

## 9 MOLECULAR TARGETS

The pathophysiology of diabetic wounds is not fully understood, but studies have shown that the dysregulation of cytokines, growth factors, and other molecular components necessary for the proper and normal healing process, as well as the unique cellular expressions of all involved cells, contribute to poor wound recovery [22]. Diabetic wound management involves the utilization of molecular factors/targets such as growth factors, clotting factors, free radicals, MMPs, cytokines, nitric oxide, signaling pathways such as stromal cell-derived factor (SDF-1α), gangliosides, neuropeptides, miRNAs, HIFs, MIF, thymosin beta 4, HSP protein, substance P, and RANKL [91]. These substances affect vascularization, re-epithelialization, matrix remodeling, migration, and proliferation of ECM either directly or indirectly. These can all be impaired in wounds caused by diabetes [145].

Additionally, absorbable biomaterials, cell regeneration therapy, growth factors, and other biological targets for the management of wounds [146]. The IL family, TNF-α family, PDGF, CTGF, FGF family, VEGF, GM-CSF, and PDGF facilitate the augmentation of the inflammatory response and tissue debridement [107]. Several agents can indirectly impact molecular targets by regulating or down-regulating the expression of cells involved in healing, promoting angiogenesis based on the specific target.

Studies conducted in mice without diabetes have also demonstrated that diabetic mice's macrophages phagocytose cell debris more slowly and inefficiently than their counterparts without the disease [147]. Additionally, during the inflammatory phase, pro-inflammatory cytokines are significantly upregulated. Evidence from diabetic animals has demonstrated that Diabetic animals show dysregulated production of MCP-1 and MIP-2, linked to prolonged and enhanced infiltration of neutrophils and macrophages into wounds [148].

## 10 FUTURE THERAPEUTIC STRATEGIES

Individuals with diabetes or diabetic wounds deal with many other health complications because of critical factors like peripheral artery disease, peripheral neuropathy, and DFUs. According to numerous reviews of the literature on diabetic wound healing, it has been found that research is emerging to a greater extent in the direction of novel therapeutic strategies rather than traditional ones.

### 10.1 Understanding Recurrence and Immune Responses

Recurrence of diabetic ulcers is a frequent phenomenon, as systemic and local factors act together to prevent chronic wound healing. Systemically, persistent hyperglycemia promotes chronic low-grade inflammation, impairs angiogenesis, and affects the remodeling process of the extracellular matrix [149, 150]. Other comorbidities include neuropathy and peripheral artery disease, which also contribute to reinjury and recurrence [151]. Local factors such as sustained inflammation and immune response dysregulation are important. For example, the imbalance between pro-inflammatory and anti-inflammatory macrophages are M1 and M2, respectively-fails to resolve inflammation and hence impairs wound closure. Excessive NETs and high activity of MMP degrade ECM and essential growth factors, further hindering repair processes [152-154].

These immune responses play a critical role in the diabetic wound fate, helping to decide between effective closure or the chronic, recurring state. Lack of modulation of inflammation and lack of promotion of appropriate immune resolution are one of key reasons current therapies are less effective than needed.

### 10.2 Need for Cell and Growth Factor Therapies

The conventional management of wounds with debridement, infection control, and offloading has symptomatic relief only, failing to address the very pathophysiological mechanisms involved in the healing of diabetic wounds. Cell-based therapies (such as mesenchymal stem cells) and growth factors (such as vascular endothelial growth factor and platelet-derived growth factor) are promising approaches because they address angiogenesis deficiencies, modulate immune responses, and promote ECM remodeling, which are key to sustained wound healing.

The emerging therapies, including cell-based therapies such as MSCs and growth factor therapies including VEGF and EGF, act by directly targeting molecular and cellular dysfunctions in diabetic wounds. Such therapies offer:

- **Angiogenesis Stimulation:** Growth factors such as VEGF and PDGF act to increase the formation of blood vessels and, therefore, provide an improved amount of oxygen and nutrient delivery to the wound site [103].
- **Modulation of Immune Responses:** Cell-based therapies, by the restoration of the M1/M2 macrophage balance, have an overall role in mitigating chronic inflammation and accelerating tissue repair [155].
- **Improvement in Remodeling of ECM:** Growth factors and stem cells enhance the deposition of ECM, the formation of granulation tissue, and re-epithelialization [156].

Advanced therapies have the potential to reduce recurrence and improve the long-term outcome since they will be dealing with the root causes.

Currently, researchers are examining potential future therapeutic techniques for diabetic wounds, including stem cell therapies, platelet-rich plasma, recombinant growth factors, MMP inhibitors, substance P, and natural product-based treatments [22]. The reduced effectiveness of current management strategies for diabetic wounds stems from their inability to address the complex and varied nature of the condition. While these therapies focus on symptomatic relief, such as infection control, debridement, and offloading, they do not adequately address the underlying pathophysiological mechanisms that lead to insufficient wound healing and recurrence. Systemic complications of diabetes, such as neuropathy and vascular insufficiency, are often poorly managed, leading to an increased risk of reinjury and chronic conditions. Addressing these challenges requires the development of innovative therapies that go beyond symptomatic management to directly target the molecular and cellular mechanisms involved in wound repair [151-188]. The clinical need suggests that stimuli-responsive and multifunctional treatment techniques that might speed up diabetic wound management are likely to be a significant aspect of the future diabetic wound therapy [157]. Some preclinical studies for diabetic wound healing are listed in Table **1**.

### 10.3 Growth Factor-based Therapies

During the process of wound healing, growth factors like KGF-2, EGF, PDGF, and bFGF are engaged in nearly every stage of wound repair. This therapy has been proven effective in treating wounds and has shown promising results in animal models for diabetic wound repair [179, 180]. Only one topical growth factor-based medication, Becaplermin (0.01% Regranex^®^ gel), has received approval from the US Food and Drug Administration as effective for promoting DFU healing [181]. The advancement of growth factor delivery techniques will make it a promising treatment approach for diabetic wound care.

### 10.4 Gene-based Therapy

A promising method for treating diabetic wounds is gene therapy, a significant healthcare challenge. It involves delivering genes that encode growth factors like VEGF and PDGF to enhance wound healing [138]. Viral vectors, particularly replication-defective adenoviruses and non-viral vectors, have been widely utilized for gene delivery, showing effectiveness in delivering PDGF and VEGF to wound sites [182]. Patients with diabetes and neuropathic ulcers have shown full ulcer healing and a rapid decrease in lesion size when bovine collagen gel containing adenovirus expressing PDGF is applied [183]. Recombinant human growth hormone was produced by Morgan *et al.* using retrovirus-mediated gene transfer in athymic mice, demonstrating that grown keratinocytes restored normal epidermis [184]. Gene regulation offers another avenue for therapeutic intervention, modifying gene expression or blocking harmful pathways to enhance tissue repair. Gene therapy presents a promising approach for treating diabetic wounds by delivering growth factors with a customized approach.

### 10.5 Stem Cell-based Therapy

Stem cell therapy is being explored as a possible treatment option for diabetic wounds because of its ability to differentiate into diverse tissue types, demonstrating its encouraging outcomes in clinical trials and experimental animal research [23]. Stem cell injections, such as bone marrow mesenchymal stem cells (BM-MSCs), bone marrow-derived mononuclear cells, or peripheral blood mononuclear cells (PB-MNCs), have shown significant improvements in the healing of wounds and ischemia in people with diabetes through injectable autologous procedures [185, 186]. Studies revealed that Acrylate hyaluronic acid (AHA) hydrogels are used to treat type 1 diabetes wounds employing pluripotent stem cells (hiPSCs) and promote skin wound healing through the addition of Gingival mesenchymal stem cells (GMSCs) with silk hydrogel and chitosan [187]. Stem cells can additionally aid in the healing of wounds by secreting several bioactive compounds, including growth factors, which play an essential role in tissue restoration and organ function [188]. Research also confirmed that by promoting neovascularisation and modulating receptor pathways, EGF-stimulated MSCs can improve blood flow in diabetic mice [189]. Stem cell therapy, with its rapid advancement in the subject of diabetic wound healing, might have undergone a revolution in recent years.

## 11 SENESCENT DRIFT AND ITS CONSEQUENCES ON CELL THERAPIES

While cell therapies offer potential benefits in treating chronic wounds, they are not universally successful. One of the key reasons is the phenomenon of senescent drift, where transplanted or resident cells exhibit premature aging due to exposure to the inflammatory and oxidative stress conditions prevalent in diabetic wounds. Chronic hyperglycemia and a pro-oxidative environment in diabetic patients accelerate cellular senescence, especially in endothelial cells, fibroblasts, and keratinocytes. Senescent cells secrete pro-inflammatory cytokines and matrix-degrading enzymes-collectively known as the senescence-associated secretory phenotype (SASP), which further promote local inflammation and impair tissue repair. Poorly prepared or expanded stem cells for therapy may introduce senescent cells into the wound environment, further amplifying inflammation and impeding healing [190].

## 12 STRATEGIES TO OVERCOME SENESCENT DRIFT

Studies have also indicated that cell-based therapies alone may not lead to successful outcomes in chronic wounds, particularly in diabetic foot ulcers, due to challenges in cell survival, differentiation, and integration with host tissues [191, 192]. This highlights the necessity of optimizing cell processing methods to prevent senescence and enhance therapeutic efficacy. A number of strategies are under investigation to further improve the efficacy of cell-based therapies and to minimize the impact of senescent drift:

- **Stem Cell Preconditioning:** Further optimization of the microenvironment while culturing the cells will reduce senescence and thus optimize the therapeutic potency, such as hypoxic conditions or cytokine priming [193, 194].
- **Combination Therapies:** Stem cells work in combination with senolytics-these are agents that selectively clear senescent cells-or cytokine inhibitors to help neutralize the harmful effects of SASP [195].
- **More Sophisticated Biomaterials:** The utilization of stem cells in combination with biocompatible scaffolds or hydrogels would facilitate better survival of cells, allow for more target-specific delivery of the cells, and reduce the chance of cellular senescence [196].

### 12.1 Innovative Approaches

Gene modification by various gene-editing techniques, including CRISPR-Cas9, is also under investigation for the purpose of altering the properties of stem cells and enhancing their resistance to oxidative stress and senescence. Stem cells combined with growth factors, such as VEGF or FGF, have also shown synergistic effects in preclinical studies by improving angiogenesis, ECM remodeling, and overall wound healing outcomes [197-199].

### 12.2 Platelet-rich Plasma Therapy

Platelet-rich plasma (PRP) is a long-standing treatment for wound healing, particularly for DFUs and other types of wounds. The autologous PRP gel, containing cytokines, growth factors, chemokines, and a fibrin scaffold, promotes normal wound healing responses. Platelet alpha granules, rich in growth factors like PDGF, VEGF, and TGF-β3, facilitate cell proliferation and tissue formation [177]. A clinical study demonstrated that thrombin and chloride-activated autologous PRP gel effectively accelerates wound healing in clean DFU patients [200]. Platelet products are more acceptable and effective in stimulating healing in patients with DFUs compared to standard saline dressings [201]. Advancements in this treatment might be a promising approach for treating diabetic wound care.

### 12.3 MCs Stabilizers and Cytokine Inhibitors

Pro-inflammatory cytokines such as IL-1 are produced by M1 macrophages, which maintain a pro-inflammatory characteristic in diabetic wounds. Blocking the IL-1β pathway promotes pro-inflammatory change to anti-inflammatory macrophages, which enhances growth factor synthesis and wound healing in diabetic mice [178]. Further clinical research is needed to evaluate the safety and efficacy of cytokine inhibitor therapies. Mast cell degranulation in diabetic mice delays vascular regression, affecting angiogenesis and remodeling phases, as they release potent angiogenic agents like FGF, VEGF, and TGF-1 [85]. Photobiomodulation, when combined with human bone marrow MSCs, significantly reduces the number and degranulation of MSCs, thereby improving wound healing in diabetes [202]. Another study found that omega-3 fatty acids alter the concentration of microcytic cells, which significantly decreases the diabetic wound area [177]. Thus, mast cell stabilizers could be a promising therapeutic strategy for diabetic wound healing.

### 12.4 MMP Inhibitors

Most studies suggest that chronic healing of DW or DFUs is associated with MMP overexpression, leading to the development of MMP inhibitors (MMPIs) for DW or DFU treatment. TIMPs inhibit the elevated levels of MMPs, and their imbalance results in poor wound healing in diabetic conditions [142]. One finding confirms that (R)-ND-336, a selective MMP-9 inhibitor, significantly improves wound healing in diabetic mice by lowering macrophage infiltration and raising the angiogenesis marker VEGF [176]. The advancement of inhibiting various MMP subtypes could potentially lead to promising therapeutic advancements for diabetic wound treatment in the future.

### 12.5 Hyperbaric Oxygen Therapy

Hyperbaric oxygen is utilized in wound management to stimulate angiogenesis and promote the development and multiplication of fibroblasts [188]. Recent studies show that hyperbaric oxygen therapy (HORT) effectively treats diabetic ulcers and wound healing [203]. A diabetic mouse model showed enhanced wound healing and reduced MMP-9 levels by treating with HBO [174]. A small-scale clinical study reveals that HBO2 treatment in DFU patients enhances angiogenesis, VEGF, IL-6, adiponectin, fibrosis, and insulin-like growth factor binding protein 3, reduces ulcer size, reduces infection signs, and also induces HIF-1a and nuclear factor (NF)-κB translocation [204]. According to the findings, it suggests that diabetic foot ulcers and other chronic lesions may benefit from hyperbaric oxygen therapy.

## 13 CLINICAL TRIALS

Clinical trials are crucial for improving diabetic wound healing treatments. These studies assess the efficiency and safety of various interventions, including novel wound dressings, advanced biomaterials, stem cell therapies, and targeted drug delivery systems. These trials aim to revolutionize wound management and improve the standard of living for millions of patients globally, demonstrating the potential of advanced approaches in addressing this global health challenge. The treatment of diabetic wounds has shown significant promise using stem cell therapy, which uses angiogenesis, immunological regulation, and collagen remodeling to expedite wound healing. The ability of mesenchymal stem cells (MSCs), derived from bone marrow or adipose tissue, to differentiate into multiple cell lineages and release growth factors and cytokines makes them highly interesting. Numerous delivery methods, including direct application onto wounds and intramuscular injections, have been demonstrated in randomized controlled trials (RCTs) to enhance clinical outcomes. There are still some significant limitations, though. Small sample sizes, short follow-up periods, and inconsistent study techniques limit the generalizability of the findings. The variability in stem cell activity based on their environment and the absence of multicenter trials with uniform protocols are further challenges. To ensure long-term effectiveness and consistent treatment procedures for broader clinical use, future studies should focus on improving cell sources, delivery methods, and large-scale randomized controlled trials [205]. The general summary of the clinical trial conducted for diabetic wound healing/DFU treatment is provided in Tables **2** and **3**.

## CONCLUSION

In recent years, research on diabetic wound treatment has increased significantly. Diabetes mellitus, characterized by hyperglycaemic conditions, is a significant cause of patients' delayed wound healing. Several findings suggest that diabetes can cause delayed healing due to a variety of causes, which include reduced angiogenesis resulting in reduced vascularity and capillary density. High oxidative stress levels contribute significantly to diabetic complications and poor recovery. Increased production of ROS within cells due to high glucose levels in diabetic conditions triggers intracellular metabolic pathways, including the advanced glycation end product (AGE) production, neuropathy, and the hexosamine, polyol, and protein kinase C (PKC) pathways, which suppress antioxidant enzymes. Other factors like tissue repair and wound healing involve various processes, and dysregulation of these factors often results in impaired healing. Clinical trials are crucial for improving diabetic wound healing treatments, which evaluate the effectiveness and safety of interventions like novel wound dressings, advanced biomaterials, stem cell therapies, and targeted drug delivery systems. Recent studies have focused on combinational approaches, which have outperformed conventional ones. Combination approaches can accelerate wound healing, but further studies are needed to identify agents at different healing phases and improve clinical methodologies. Recent research is focusing on novel therapeutic strategies for diabetic wounds, including anti-inflammatory drugs, cytokine modulators, MMP inhibitors, growth factors, dual growth factors, ECM stimulators, angiogenesis stimulators, and stem cell therapies, gene-based therapies, and natural, platelet-rich plasma, MCs Stabilizers and Cytokine inhibitors, substance P, and natural product-based treatment., have been tested with mixed results. Recent advancements in diabetic wound healing mechanisms have improved diagnosis and treatment, but there are still unmet needs in clinical wound management. As diabetic patient populations increase, their management becomes crucial, necessitating further research to improve patient quality of life. The recurrence of diabetic ulcers and the limited management strategies raise an urgent call for new therapies that address some underlying pathophysiological mechanisms. Advanced strategies, including cell-based therapies and growth factor treatments, seem very promising for targets such as sustained inflammation, crippled angiogenesis, and ECM dysregulation. Challenges ahead include senescent drift and suboptimal efficacy of single-agent therapies. Strategies such as cell preconditioning, senolytic combination therapy, and use of biomaterial scaffolds point to a way forward. The development of therapies that are multifunctional and driven by mechanisms of action will be critical in the improvement of outcomes and quality of life of affected individuals as the prevalence of diabetes continues to rise. Further research is needed to discover agents at various phases of diabetic wound management, and better clinical techniques and systems can aid in recognizing the level of healing.

## Figures and Tables

**Fig. (1):**
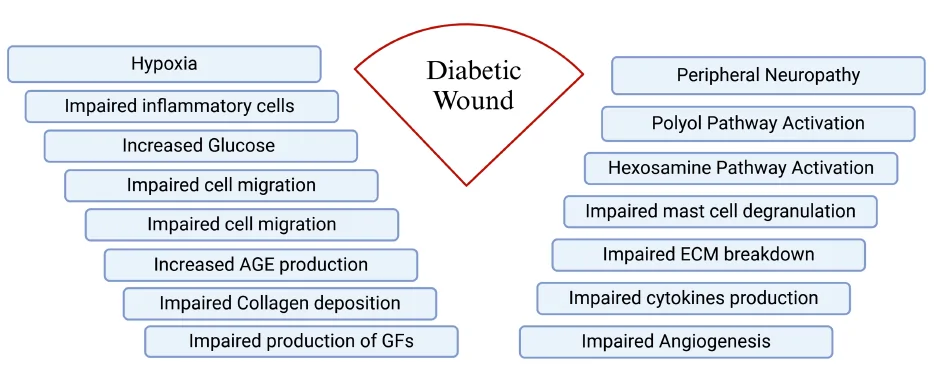
Key factors influencing diabetic wound healing.

**Fig. (2):**
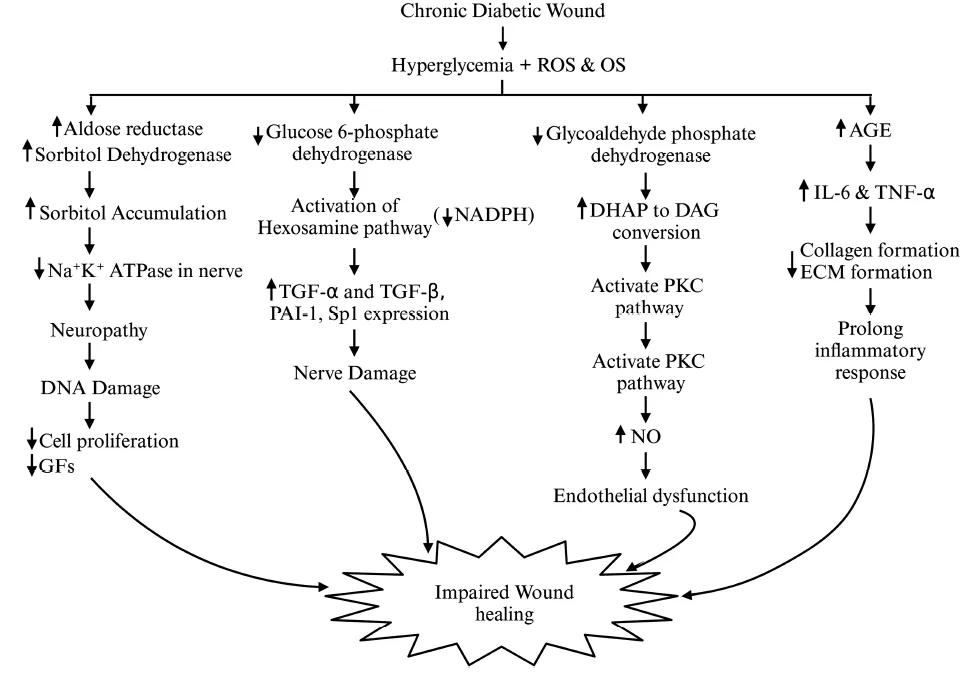
Roles of free radicals and oxidative stress in diabetic wound.

**Fig. (3):**
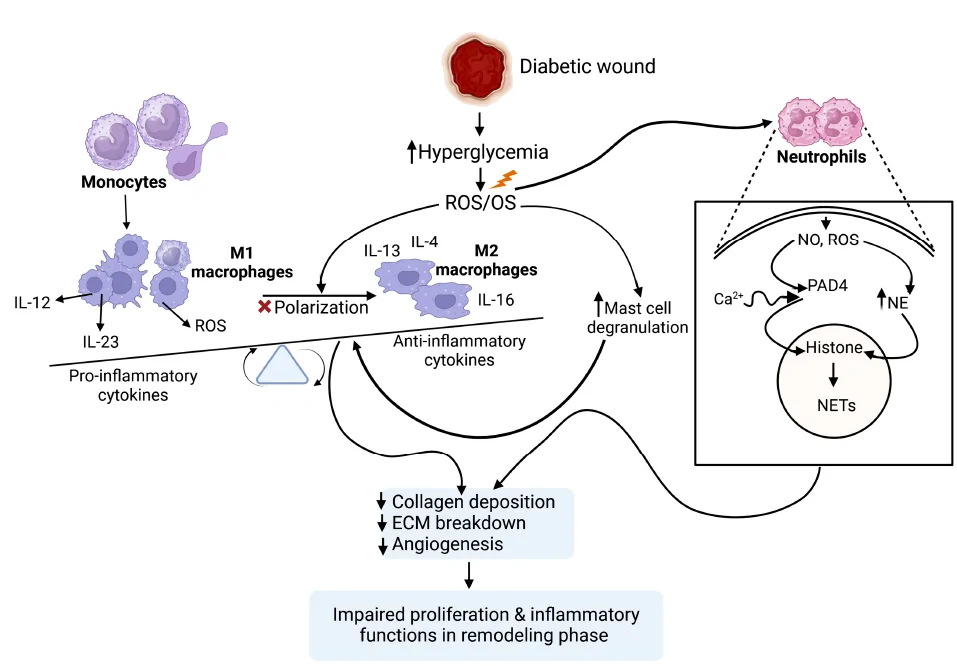
Mechanism and impacts on inflammation, cellular dynamics, and tissue repair in diabetic wound.

**Fig. (4):**
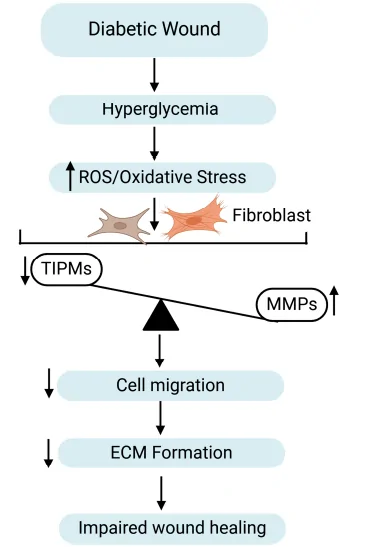
The role of matrix metalloproteinases (MMPs) in diabetic wound healing.

**Table 1:** Pre-clinical studies of treatment for diabetic wound.

**S. No.**	**Therapeutic Agent**	**Animal Study Models**	**Route of Administration**	**Dose**	**Wound Size**	**Outcomes**	**References**
1	VEGF	Diabetic db/db mice	Intradermal injection	4 mg (5.04 μg) in 500 μl PBS	6- and 8-mm diameter	↑ formation of collagen, granulation tissue, dermal remodeling, and upregulated VEGFR2 expression	[158]
2	Plasmid-VEGF	Streptozotocin-induced diabetic mice	Subcutaneous injection	20 μg	6 mm diameter, circular	↑ wound closure time, increased skin blood perfusion, and CD31 expression	[159]
3	Adenovirus encoding VEGF	Genetically diabetic mice	Intradermal Injection	5 × 1010, 5 × 1011, 1.6 × 1010, 1.6 × 1011 vp per wound	1.4 cm diameter, circular	↑ re-epithelialization, vascularized granulation tissue formation, and more organized collagen fiber bundles, and wound closure by 27 days	[160]
4	VEGF- and bFGF	Diabetic db/db mice	Topically	300 μg of VEGF and 300 μg bFGF	8 mm thickness	↑ rate of wound closure, re-epithelialization, mature granulation tissue, collagen deposition	[161]
5	rhPDGF	Diabetic Wistar rats	Topically	7.0 mg/cm^2^	2.54 cm^2^	↑ proliferation, re-epithelialization, organized granulation tissue formation, and enhanced c-Fos protein expression by activating the ERK pathway	[162]
6	rhEGF	Diabetic db/db mice	Topically	1 and 10 μg/ml	10×10 mm^2^	wound re-epithelialization, granulation tissue score, craniocaudal contraction, and angiogenesis	[105]
7	IGF-1	Alloxan-induced male Wistar rats	Topically	1% IGF-1	0.9 mm diameter	↑ re-epithelialization, formation of collagen, and granulation tissue ↓inflammatory cells ↑ The expression of myofibroblasts	[163]
8	rhKGF-1	Streptozotocin induced Wistar rats	Topically	50 ng/ml	1 cm^2^	↑ cell migration, wound contraction, and promote healing; ↑ TGF-β1, p-Smad2, and p-Smad3 expressions	[164]
9	bFGF	Sprague-Dawley rats	Topically	12.5 mg	250 mm^2^	↑ migration, proliferation, re-epithelialization, angiogenesis and ECM remodeling	[165]
10	TGF-α	Alloxan-induced adult male rats	Intravenous injection	500 ng/kg	1 cm length and 1/2 cm depth	Showed almost complete re-epithelialization, well-organized granulation tissue, ↑ collagen bundles, and endothelial cell proliferation	[166]
11	EGF	High-fat/high-sucrose diet- and alloxan-induced and diabetic New Zealand rabbits	Topically	100 mg/l	10×10 mm	↑ wound healing rates, granulation tissue production, neutrophil numbers, collagen uniformity, ECM deposition, number of capillaries and fibroblasts	[167]
12	PRP hydrogel	Streptozotocin-induced Sprague Dawley rats	Topically	1 mL	5 mm diameter	↑ collagen deposition, angiogenesis, and nerve repair	[168]
13	PRP	Streptozotocin-induced diabetic rats	Topically	-	1 cm diameter	Significant decrease in wound size and increases the EGF, FGF-2, TGF-β1, and VEGF-α expression levels	[169]
14	Amniotic mesenchymal stem cell	NOD)/SCID mice	Intradermal injection	1 × 10^6^ cells suspended in 100 µL PBS	6 mm full-thickness excision	↑ expression of IGF-1, EGF, and IL-8	[170]
15	Placenta mesenchymal stem cell	Diabetic Goto Kakizaki rats	Intradermal injection	10^2^ cells in 200 µl PBS	8 mm diameter	Enhanced healing by promoting vascular regeneration	[171]
16	Adipose-derived stem cells	Diabetic Sprague Dawley rats	Injection	2.5 × 106 cell mL^-1^	1.1 cm diameter	↑ angiogenesis and re-epithelialization ↓ inflammation	[172]
17	Adipose-derived stem cells, Endothelial-differentiated stem cells	Diabetic swine	Injection	5 and 10 million ASC cells	5 cm circular full-thickness excision	The wound closure rates in cell-based treatments have seen an increase and showed enhanced angiogenesis	[173]
18	Hyperbaric Oxygen (HBO) Therapy	Diabetic db/db mice	HBO chamber	50 to 250 psig	8 mm diameter	↓levels of active MMP-9	[174]
19	MMP-9 Inhibitor	Diabetic db/db mice	Topically	200 μg/mL	8 mm diameter	↓ levels of MMP-8 and MMP-9	[175]
20	Broad-spectrum MMP inhibitor	Diabetic db/db mice	Topically	5 mg/mL	_	↑ healing rate, re-epithelialization and inhibit the activity of MMP-9	[176]
21	Omega-3 (Omegaven)	Streptozotocin-induced diabetic rats	Intraperitoneally	1mL	20 mm× 20 mm	↑ wound closure rate and a significant decrease in grade 3 mast cells	[177]
22	IL-1β-neutralizing antibody	Diabetic db/db mice	Intradermal injection	20 μg	8 mm diameter	↑ re-epithelialization and formation of granulation tissue, ↑ IGF-1 and TGF-b levels ↓ IL-1 and TNF-α levels	[178]

**Table 2:** Clinical studies of growth factor-based therapy for diabetic wound.

** S. No **	** Therapeutic Agents **	** Trial Methods **	** Participants **	** Administration **	** Dose and Duration **	** Outcome **	** Country of Study **	** References **
1	Recombinant human epidermal growth factor (hEGF)	Stratified randomized open-labeled controlled trial	73 participants	Cream applied locally	Regen-D 150 once daily for 30 days	The ulcer healed in 78% of treated patients	India	[205]
2	Recombinant Epidermal growth factor and acidic fibroblast growth factor	Randomized controlled	199 participants	Applied topically	40 IU/cm ^ 2 ^ of hEGF and aFGF once per day for 60 days	Shorter wound healing duration, and significant Granulated tissue maturation in a short time	China	[206]
3	Basic fibroblast growth factor	Randomized, double-blind, dose-ranging, placebo-controlled trial	150 participants	Topical spraying	50 μg of bFGF and 50 μg of bFGF once a day for 8 weeks	Significantly reduces ulcer area by 72.3% and 82.2% with bFGF and bFGF, respectively	Japan	[207]
4	Recombinant human platelet-derived growth factor	Randomized double-blind trial	50 participants	Gel applied topically	rhPDGF 0.01% once daily for 10 weeks	No significant changes observed	India	[208]
5	Recombinant human vascular endothelial growth factor	Phase I double-blind placebo-controlled randomized trial	55 participants	Applied topically	72 µg/cm^2^ three times a week for 12 weeks	Improved the incidence of complete wound healing	United States	[209]
6	Platelet-derived growth factor gene-activated matrix	Open-label, uncontrolled, dose-escalation Phase 1/2 study	15 participants	_	11010, 31010, and 11011 vp/cm^2^ for 12 weeks	Significantly decrease the ulcer size in most of the patients by the end of 3 months	United States	[188]

**Table 3:** Clinical studies of cell-based and other therapy for diabetic wound.

** S. No **	** Therapeutic Agents **	** Trial Methods **	** Participants **	** Administration **	** Dose and Duration of study **	** Outcome **	** Country of Study **	** References **
	** A. Cell-Based Therapy **							
1	Autologous platelet-rich plasma gel	Single-arm clinical trial	100 participants	Gel applied topically	2mL/cm ^ 2 ^ for 4 weeks	Significantly decreased wound area compared to the control group.	Iran	[210]
2	Platelet-rich plasma	Prospective randomized controlled trial	20 participants	Intradermal injection	1 mL	PRP-treated group experienced an 8-week faster healing time compared to the control group.	India	[211]
3	Autologous mesenchymal stromal cell therapy	Clinical case study	3 participants	Applied topical	1 × 106 cells/cm ^ 2 ^	Low doses of MSCs improve the re-epithelialization of DFU	Russia	[212]
4	Allogeneic adipose-derived stem cell	Randomized, comparator- controlled, single-blind, parallel-group, multicenter study	59 participants	Hydrogel applied topically	1 × 10 ^ 6 ^ cells/sheet for 12 weeks	82% of the treated group reached complete wound closure at week 12, while 53% were in the control group.	Korea	[213]
5	Bone marrow mononuclear cells	Prospective, controlled, randomized trial	30 participants	Intramuscular or intraarterial injection	1 mL for 52 weeks	This stem cell therapy could improve microcirculation, thus potentially supporting wound healing in diabetic foot patients without the option of revascularization.	Germany	[214]
6	G-CSF-mobilized peripheral blood CD34+ cells	Phase I/IIa prospective study	5 participants	Intramuscular injection	2 × 10 ^ 7 ^ CD34 ^ + ^ cells/patient for 12 weeks	Complete wound closure was observed at an average of 18 weeks with increased vascular perfusion in all patients	Japan	[215]
7	Bone marrow mononuclear cells	-	6 participants	Intralesional injection	15 mL for 6 months	All patients healed within 9 months, with an increase in the toe-brachial index and a decrease in rest pain, and an increase in pain-free walking.	India	[216]
	** B. Other Therapy **							
8	Topical oxygen therapy	Multi-centre, open, randomized controlled trial	145 participants	Applied topically	Topical oxygen therapy for 12 weeks	Significantly reduced wound area by 70% compared to the standard care group with 40%	United States	[217]

## LIST OF ABBREVIATIONS

AGE: Advanced Glycation End Product
ASC: Adipose-derived Stem Cell
ATF-3: Activating Transcription Factor 3
BM-MNC: Bone Marrow-derived Mononuclear Cell
BM-MSC: Bone Marrow Mesenchymal Stem Cell
CD31: Cluster of Differentiation 31
DAG: Diacylglycerol
DFU: Diabetic Foot Ulcers
DHAP: Dihydroxyacetone Phosphate
DM: Diabetes Mellitus
DW: Diabetic Wound
ECM: Extracellular Matrix
EGF: Endothelial Growth Factor
eNOS: Endothelial Nitric Oxide Synthase
EPC: Endothelial Progenitor Cell
ERK1/2: Extracellular Signal-Regulated Kinase 1/2
GM-CSF: Granulocyte Macrophage Colony Stimulating Factor
HIF-1: Hypoxia Inducible Factor-1
IGF: Insulin-like Growth Factor
IL: Interleukin
KGF: Keratinocyte Growth Factor
LPS: Lipopolysaccharide
MCP-1: Monocyte Chemoattractant Protein-1
MIF: Macrophage Migration Inhibitory Factor
MIP-2: Macrophage Inflammatory Protein-2
MMP: Matrix Metalloproteinase
MSC: Mesenchymal Stem Cell
NADPH: Nicotinamide Adenine Dinucleotide Phosphate Hydrogen
NE: Neutrophil Elastase
NET: Neutrophil Extracellular Trap
NGF: Nerve Growth Factor
NO: Nitric Oxide
Nrf2: Nuclear Factor Erythroid 2-Related Factor 2
PAD-4: Peptidyl Arginine Deiminase 4
PAI-1: Plasminogen Activator Inhibitor-1
PB-MNC: Peripheral Blood Mononuclear Cells
PDGF: Platelet-derived Growth Factor
PKB: Protein Kinase B
PKC: Protein Kinase C
PRP: Platelet-rich Plasma
PSIG: Pounds Per Square Inch Gauge
RANKL: Receptor Activator of Nuclear Factor Kappa-Β Ligand
RNS: Reactive Nitrogen Species
ROS: Reactive Oxygen Species
SDF-1a: Stromal Cell-derived Factor 1a
Smad: Suppressor of Mother Against Decapentaplegic
FGF: Fibroblast Growth Factor
TGF: Transforming Growth Factor
TNF-α: Tumor Necrosis Factor- α
UPR: Unfolded Protein Response
VEGF: Vascular Endothelial Growth Factor
VP: Viral Particles
